# Integrated analysis of long noncoding RNAs and mRNA expression profiles reveals the potential role of lncRNAs in early stage of post-peripheral nerve injury in Sprague-Dawley rats

**DOI:** 10.18632/aging.202989

**Published:** 2021-05-08

**Authors:** Yujing Zhang, Zhaowei Zhu, Xiangxia Liu, Shuqia Xu, Yi Zhang, Yangbin Xu, Bo He

**Affiliations:** 1 Department of Plastic Surgery, The First Affiliated Hospital of Sun Yat-Sen University, Guangzhou 510080, China; 2 Department of Orthopedics, The Third Affiliated Hospital of Sun Yat-Sen University, Guangzhou 510630, China

**Keywords:** peripheral nerve injury, long noncoding RNA, bioinformatics, lncRNA-mRNA network

## Abstract

The regulatory role of lncRNAs in the early stage post peripheral nerve injury (PNI) is not yet clear. In this study, next-generation sequencing was used to perform deep sequencing on normal sciatic nerves (control) and lesional tissues derived on the 4th (D4) and 7th days (D7) after sciatic nerve injury in rats. Time-point unique differentially expressed lncRNAs (DElncRNAs) were analyzed for functional enrichment. The results showed that 776 DElncRNAs were unique to D4, and their functions were mainly enriched in wound healing, phosphatase binding and MAPK signaling pathways; 317 DElncRNAs were unique to D7, and their functions were mainly enriched in ion transmembrane transporter channel activity; 579 DElncRNAs were shared by these two days, and their functions were mainly enriched in axongenesis, the PI3K-Akt signaling pathway and cell cycle. Furthermore, lncRNA-mRNA interaction networks were constructed in functions or pathways with a high enrichment rate. Finally, 3 mRNAs and 4 lncRNAs in the axongenesis interaction network were selected, and their expression levels were verified by RT-qPCR. This study preliminarily revealed the regulatory role of lncRNAs at different time points in the early stage post PNI, which provides potential targets for basic research and clinical treatment of PNI.

## INTRODUCTION

The morbidity of peripheral nerve injury (PNI) has been increasing with the development of social industrialization, and such an injury has a negative effect on the individual and family life, causes the loss of social labor, and requires a large amount of medical resources [1, 2]. The main clinical manifestations of the disease include peripheral sensory, motor and autonomic nerve disorders [3]. The pathophysiological changes after PNI include Wallerian degeneration of the distal stump of the injured nerve and the formation of a growth cone from the proximal stump, followed by the regenerated proximal nerve fibers growing through to the distal end and reinnervating the target organs to complete the nerve regeneration process [4]. The expression of a number of pivotal genes, enriched in the inflammatory response, immune response, cell migration, cell proliferation and other key biological processes involved in peripheral nerve regeneration was reported to reach a high plateau within one week [5]. During this regeneration and repair process, such genes undergo a complex and dynamic regulation [6]; however, to date, few studies have examined this issue. Therefore, a comprehensive understanding of the regulation rules in such a process could provide a solid foundation for the basic research and clinical application of PNI treatment.

LncRNAs are a type of noncoding RNA with a length of more than 200 nucleotides [7]. Under usual circumstances, lncRNAs do not encode proteins themselves, but they can regulate gene expression in different ways, and in turn participate in many physiological and pathological processes [8]. According to the mechanism of gene expression regulation, lncRNAs can be roughly divided into two categories: cis and trans. Cis-acting lncRNAs mainly regulate their neighboring chromatin state or gene expression, while trans-acting lncRNAs affect gene expression in remote transcripts [9].

Limited research on the regulatory role of lncRNAs in the process of peripheral nerve injury and repair has been reported thus far. Ma and colleagues found that the expression of lncRNA MEG3 was upregulated in a sciatic nerve injury model, and downregulating the expression of such lncRNA in Schwann cells can promote cell proliferation and migration and induce the growth of nerve axons [10]. Another study showed that lncRNA TNXA-PS1 could function as an endogenous competing RNA, which regulates the expression of Dusp1 protein in Schwann cells by adsorbing miR-24-3p and miR-152-3p and in turn affects the migration of Schwann cells [11]. Pan and other researchers used microarray data to analyze the regulatory relationship between differentially expressed lncRNAs and mRNAs in the early stage of mouse sciatic nerve injury. The results of the analysis suggest that JUN, a key regulatory gene after PNI, could be cis-regulated by lncRNA ENSMUSG00000087366, and the other gene MBP, mainly involved in myelination, may be trans-regulated by lncRNA ENSMUSG00000084785 [12]. However, most of the abovementioned studies were based on gene chip technology. The main disadvantage of such a technology is that it can only detect and analyze known genes and omits unknown lncRNAs that may play a role in peripheral nerve damage and repair.

In this study, we used next-generation sequencing (NGS) to perform deep sequencing of lncRNAs and mRNAs at different time points after rat sciatic nerve injury. Differentially expressed lncRNAs (DElncRNAs), unique to each time point or shared by both, were first analyzed and combined with the corresponding differentially expressed mRNA (DEG) data we predicted the cis- and trans-acting target genes of the DElncRNAs. Gene Ontology (GO) and Kyoto Encyclopedia of Genes and Genomes (KEGG) pathway enrichment analyses were then performed on these DElncRNAs, and we constructed lncRNA-mRNA interaction networks in some GO terms and KEGG pathways with a high enrichment score. Finally, the expression of 3 mRNAs and 4 lncRNAs in network axongenesis was verified by RT-qPCR. This study preliminarily revealed the potential regulatory role of lncRNAs at different time points in the early stage of PNI.

## MATERIALS AND METHODS

### Laboratory animal selection and ethics statement

**Figure 1:**
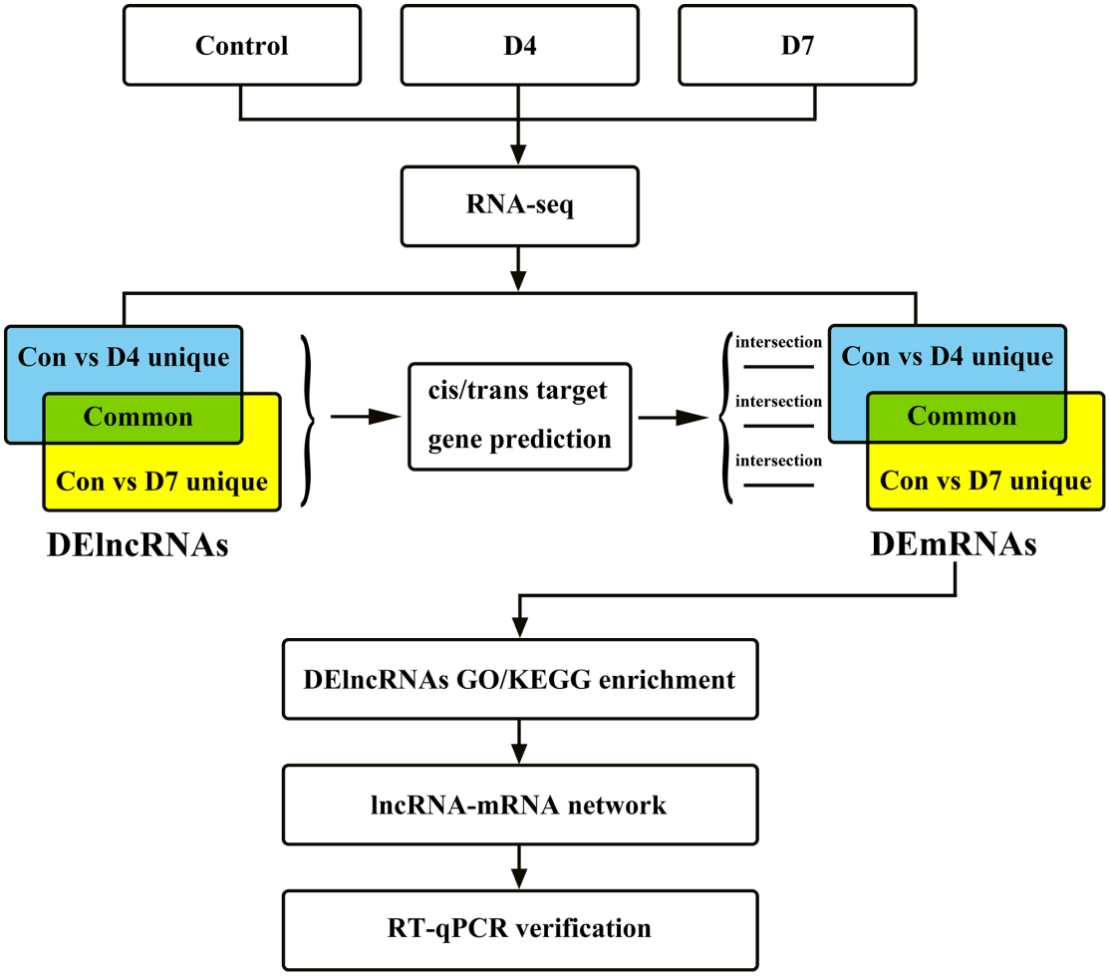
The research flow chart of the integrated analysis of lncRNAs and mRNA expression profiles in early stage of post-peripheral nerve injury in Sprague-Dawley rats.

Twenty-one 1-month-old Sprague-Dawley (SD) rats weighing approximately 150-200 g were randomly divided into 3 groups: the control group (control), 4 days after sciatic nerve injury (D4) and 7 days after injury (D7). This experiment was approved by the Experimental Animal Ethics Committee of Sun Yat-sen University. All experimental operations complied with the rules and regulations formulated by the ethics committee, and the damage and pain caused to animals were minimized during the experiment. The overall experimental design and workflow of this research are shown in Figure 1.

### Sciatic nerve clamp injury model

Animals in the experimental groups (D4 and D7) were anesthetized by intraperitoneal injection of 10% chloral hydrate (0.3 ml/100 g) and were subjected to sciatic nerve crush injury model according to our previously published articles [13, 14]. The operation is briefly described as follows. After the left hind limb and back of the rat were shaved and disinfected, the skin was cut parallel to the femur, and the superficial gluteus muscle was bluntly separated to fully expose the left sciatic nerve. The middle segment of the sciatic nerve was clamped with hemostatic forceps for 30 s and then released immediately, and the wound was closed layer by layer. Animals in the control group underwent the same operation but no sciatic nerve clamp operation. All rats were kept in a suitable and stable environment, ambient temperature between 20-25° C and humidity between 50-65%, with adequate food and water supply.

### RNA extraction and purification

Two rats were randomly selected from each group, and RNA extraction and purification were performed on either normal or injured sciatic nerve samples using the mir VanaTM miRNA Isolation Kit (Cat# AM1561, Ambion, MA, USA) according to the standard protocol. The total RNA obtained was subjected to quality inspection by electrophoresis and purification using an RNAClean XP kit (Cat# A63987, Beckman Coulter, Inc., CA, USA) and RNase-Free DNase Set (Cat#79254, QIAGEN, Duesseldorf, Germany). The purified RNA was tested for quality by NanoDrop ND-2000 spectrophotometer (Thermo Fisher, MA, USA) and Agilent Bioanalyzer 2100 (Agilent Technologies, CA, USA).

### Sequencing library construction, RNA sequencing and data analysis

Purified total RNA was subjected to rRNA removal, fragmentation, first- and second-strand cDNA synthesis, end repair, poly-A tail addition, ligation, enrichment and other steps to construct a sequencing sample library. High-throughput RNA paired-end sequencing was then performed using the Illumina HiSeq Xten platform (Illumina, Inc., CA, USA). The unqualified reads, with low overall or end quality and containing sequencing primers, etc. were removed from the raw reads to obtain clean reads, from which rat ribosomal RNAs were further filtered. The spliced mapping algorithm in Hisat2 (version: 2.0.4) [15] was applied to map the above processed reads to the rat reference genome (ftp://ftp.ensembl.org/pub/release-100/fasta/rattus_norvegicus/dna/).

With the use of the Stringtie software (version: 1.3.0) [16], the mapped reads were assembled to construct the transcripts, which were then matched to the reference databases (NOCODE and Ensembl) using GffCompare (version: 0.9.8) software to obtain annotation. The unmatched transcripts were subjected to potential novel lncRNA prediction with the following criteria: (1) transcript length ≥ 200 bp AND exon ≥ 2, (2) the predicted ORF < 300 bp, (3) coding potential calculator (CPC) score < 0 [17] AND coding-noncoding index (CNCI) score < 0 [18] AND with no significant difference using Pfam comparison [19]. The Perl script was then applied to locate the position of the above novel lncRNAs on the chromosomes and to make annotations.

With the use of Stringtie software, the fragment numbers of each gene were counted, which were then normalized by TMM (trimmed mean of M values) method. Finally the FPKM value of each known or newly predicted lncRNAs was calculated using perl script, where the lncRNA ID starting with NON is the known lncRNA in the NONCODE database, the ID starting with ENS is the known lncRNA in the Ensembl database, and the ID starting with MSTRG is the newly predicted lncRNA. The edgeR package [20] was then used to analyze the differentially expressed genes between samples (Control vs D4 and Control vs D7). The screening criteria for differentially expressed genes were as follows: (1) q-value (the corrected p-value) < 0.05, and (2) the absolute value of fold change ≥ 2. The RNA-seq data were released to the National Center for Biotechnology Information (NCBI) Gene Expression Omnibus (GEO) database under the accession number GSE162548.

### Differentially expressed lncRNA grouping and target gene prediction

The intersection of the DElncRNAs, compared between the control vs D4 and the control vs D7 groups, was made, and the DElncRNAs were divided into three groups according to the intersection results: (1) DElncRNAs unique to the control vs D4 group, (2) common DElncRNAs shared by the control vs D4 and the control vs D7 groups, and (3) DElncRNAs unique to the control vs D7 group. Cis- and trans-target gene prediction was then performed for the DElncRNAs in the above three groups. Target genes located with a distance less than 10 kb from the upstream or downstream of the lncRNA were considered as the cis-target gene. For trans-target gene prediction, the NCBI BLAST tool was first used to select rat mRNA sequences that had complementarity or similarity with the DElncRNA sequences, and then the RNAplex software [21] was used to calculate the complementary energy between the two sequences. Target sequences with an identity greater than 85% and an e-value greater than e-20 were selected as the trans-targets of the lncRNA. Refer to the Supplementary Tables 1, 2 for the cis- and trans-target genes, respectively.

### LncRNA functional enrichment and lncRNA-mRNA interaction network construction

According to the DElncRNAs grouping method, the differentially expressed mRNAs (DEGs, DEG screening criteria were the same as DElncRNAs), compared between the control vs D4 and the control vs D7 groups, were intersected and then divided into three groups: (1) DEGs unique to the control vs D4 group, (2) common DEGs shared by these two groups, (3) DEGs unique to the control vs D7 group. The predicted target genes of DElncRNAs intersecting with the corresponding DEGs in each group were subjected to GO functional enrichment analysis, including biological process (BP), molecular function (MF) and cellular component (CC), and KEGG pathway enrichment analysis using the Bioconductor program [22] in R software. The enrichment results with p-values less than 0.05 were selected and displayed with dot plots. The GO terms or KEGG pathways with a high enrichment score in each group were selected for lncRNA-mRNA interaction network analysis, and the results were processed and displayed with Cytoscape software.

### Real-time quantitative PCR verification

Three sets of lncRNA-mRNA interaction networks in the GO term axongenesis were selected, namely, Gndf and NONRATT015075.2, Nfasc and NONRATT008698.2, Pmp22 and NONRATT004387.2, NONRATT004386.2. The gene expression of the above 3 mRNAs and 4 lncRNAs was verified by RT-qPCR in the remaining 5 animals in each group. Reverse transcription of RNA was performed using the ReverTra Ace qPCR Kit (TOYOBO, #FSQ-101, Osaka, Japan). The reaction conditions were set as follows: 37° C, 15 min, then 98° C, 5 min, and the obtained cDNA was stored at 4° C. The PCR system was prepared according to the Power SYBR Green PCR Master Mix (ABI, #4368708, MA, USA) kit operation manual and then subjected to RT-qPCR in the QuantStudio 5 Real-Time PCR System (ABI) instrument. The reaction conditions were set as follows: 50° C, 2 min, then 95° C, 10 min, and then 40 reaction cycles of 95° C, 15 s and 60° C, 1 min. Refer to Supplementary Table 3 for the PCR primer sequences.

### Statistical analysis

The RT-qPCR data were statistically analyzed by R software, and the statistical results are expressed as the mean ± standard error (SEM). If the data of two independent samples were content with normal distribution and the variances were uniform, then Student’s t-test was used to analyze the differences between these two samples. If the variances were not uniform, the t-test with Welch’s correction was used for analysis. If the data were not normally distributed, the Wilcoxon test was used for analysis. The results were displayed using R software and GraphPad Prism software (version: 8.4.3). A p-value less than 0.05 was considered statistically significant, such as *P < 0.05, **P < 0.01, ***P < 0.001. The RT-qPCR statistical results were all from 5 independent experiments.

## RESULTS

After completing the construction of the cDNA library of the sequencing samples, RNA sequencing was performed using the Illumina HiSeq Xten platform. The number of raw reads in each group and subsequent processed data are shown in Table 1. Some unqualified reads with low overall quality, sequencing primers, low end quality, etc., were removed to obtain clean reads. The results showed that the clean reads accounted for a high proportion of the raw reads (94.76%-96.43%), indicating the high quality and reliability of our sequencing data. Ribosomal RNA reads and reads with only single-end sequencing results were further removed, and the remaining reads (pre-mapped reads) were matched to the rat genome sequences to obtain the mapped reads. The mapping rate of all the 6 groups reached a percentage greater than 90%, except for the Con_a group (88.70%). The unique mapped reads ratio of each group was above 99%.

**Table 1:** The preprocessing results of RNA-seq raw data.

**Samples**	**Raw reads**	**Clean reads**	**Pre-mapped reads**	**Mapped reads**	**Unique mapped reads**
Con_a	129,843,600	125,210,144 (96.43%)	123,607,746	109,637,530 (88.70%)	109,298,743 (99.69%)
Con_b	115,591,386	111,263,970 (96.26%)	109,578,988	99,997,376 (91.26%)	99,696,461(99.70%)
D4_a	127,454,796	120,772,863 (94.76%)	118,057,468	107,438,995 (91.01%)	106,994,858 (99.59%)
D4_b	139,487,928	133,447,705 (95.67%)	130,573,342	118,971,351 (91.11%)	118,531,648 (99.63%)
D7_a	122,870,410	118,282,583 (96.27%)	115,539,970	105,751,413 (91.53%)	105,400,258 (99.67%)
D7_b	116,964,418	112,154,519 (95.89%)	109,614,408	100,594,290 (91.77%)	100,226,082 (99.63%)

Clean reads ratio = Clean reads/Raw reads; Mapped reads ratio = Mapped reads/Pre-mapped reads; Pre-mapped reads = Clean reads - rRNA reads – single-end reads; Unique mapped reads ratio = unique mapped reads/mapped reads.

### LncRNA characteristics analysis

**Figure 2:**
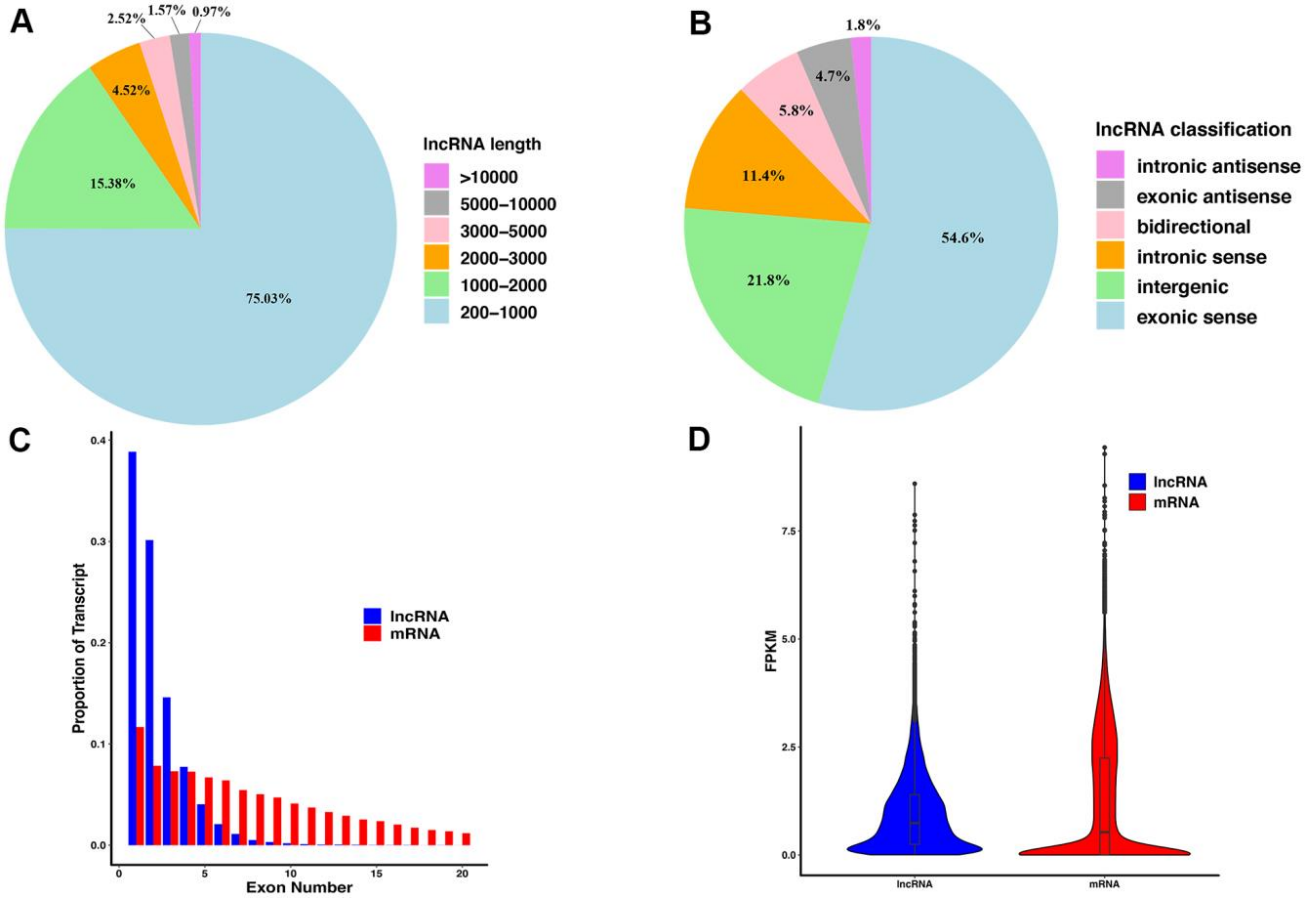
**The features of lncRNAs derived from injured sciatic nerves.** (**A**) The length distribution of lncRNAs. (**B**) The classification of lncRNAs. (**C**) Exon number distribution per transcript of lncRNAs and mRNA. (**D**) The FPKM of lncRNAs and mRNA.

The lncRNAs identified by sequencing were classified and counted according to their lengths (Figure 2A). As the length of lncRNAs increases, its proportion to the total lncRNA number decreases. LncRNAs with a length of 200 - 1,000 nt accounted for the highest proportion, up to 75.03%, and lncRNAs with a length greater than 10,000 nt accounted for the lowest proportion, only 0.97%. Furthermore, lncRNAs were classified according to their location in the genome (Figure 2B), and the results showed that the lncRNAs originating from the exon region of the coding sequences (exonic sense) accounted for the highest proportion (54.6%), followed by the intergenic lncRNAs (21.8%). The lncRNAs originating from the intron region of the antisense strand (intronic antisense) accounted for the lowest proportion (1.8%). The lncRNAs and mRNAs were then statistically analyzed according to the exon numbers (Figure 2C), and the results showed that lncRNAs containing only one exon accounted for the largest proportion (38.86%), and the proportion of lncRNAs containing 1 to 4 exon numbers to the total number of lncRNAs was over 91%. Such results were consistent with the lncRNA length proportion: the proportion of lncRNAs less than 2,000 nt was 90.41%. Finally, the average expression value of lncRNAs and mRNAs was analyzed (Figure 2D): The average expression level of lncRNAs is slightly higher than that of mRNAs, and the expression value distribution is more concentrated.

### DElncRNAs analysis and grouping

**Figure 3:**
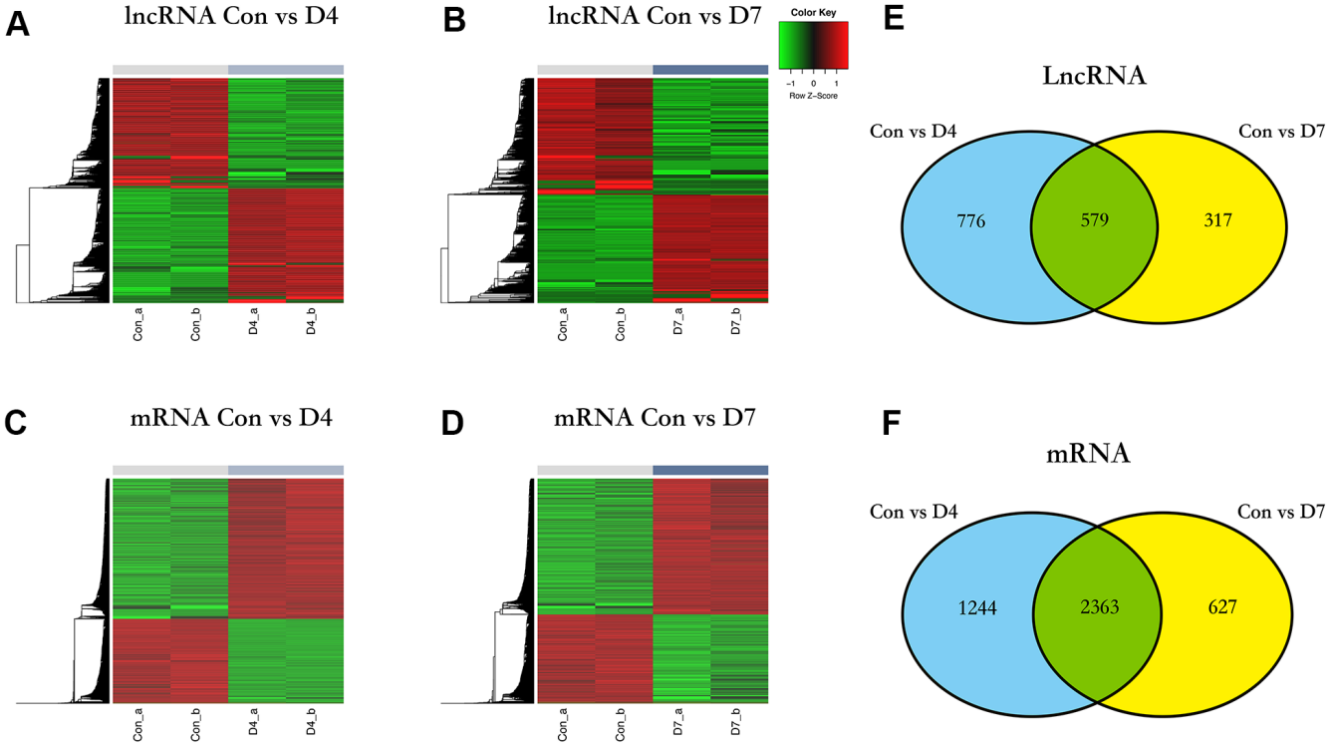
**The differentially expressed lncRNAs and mRNA in different time points post nerve injury.** (**A**) Heatmap of lncRNAs expression in control and D4 group. (**B**) Heatmap of lncRNAs expression in control and D7 group. (**C**) Heatmap of mRNA expression in control and D4 group. (**D**) Heatmap of mRNA expression in control and D7 group. (**E**) The intersection of differentially expressed lncRNAs between the control vs D4 and control vs D7 groups. (**F**) The intersection of differentially expressed mRNA between the control vs D4 and the control vs D7 groups.

The expression values of lncRNAs on D4 and D7 post-nerve injury were compared to the normal tissue (control vs D4 and control vs D7). In the control vs D4 group, a total of 1355 DElncRNAs were identified, of which 691 were upregulated and 664 were downregulated (Figure 3A), and in the control vs D7 group, a total of 896 DElncRNAs were identified, of which 431 were upregulated and 465 were downregulated (Figure 3B). The same screening criteria were applied to identify the DEGs: in the control vs D4 group, a total of 3607 DEGs were identified, of which 2240 were upregulated and 1367 were downregulated (Figure 3C), and in the control vs D7 group, a total of 2990 DEGs were identified, of which 1798 were upregulated and 1192 were downregulated (Figure 3D).

To further investigate the role that lncRNAs play at different time points in the early stage of post-sciatic nerve injury, we intersected the DElncRNAs of control vs D4 and control vs D7 groups and identified some differences and similarities: there are 776 DElncRNAs unique to the control vs D4 group, 317 DElncRNAs unique to the control vs D7 group, and 579 common DElncRNAs shared by these two groups. The obtained data were sorted to prepare for the subsequent functional enrichment analysis (Figure 3E), and the unique and common DEGs were analyzed and sorted using the same method (Figure 3F).

### GO and KEGG pathway enrichment of the DElncRNAs

**Figure 4:**
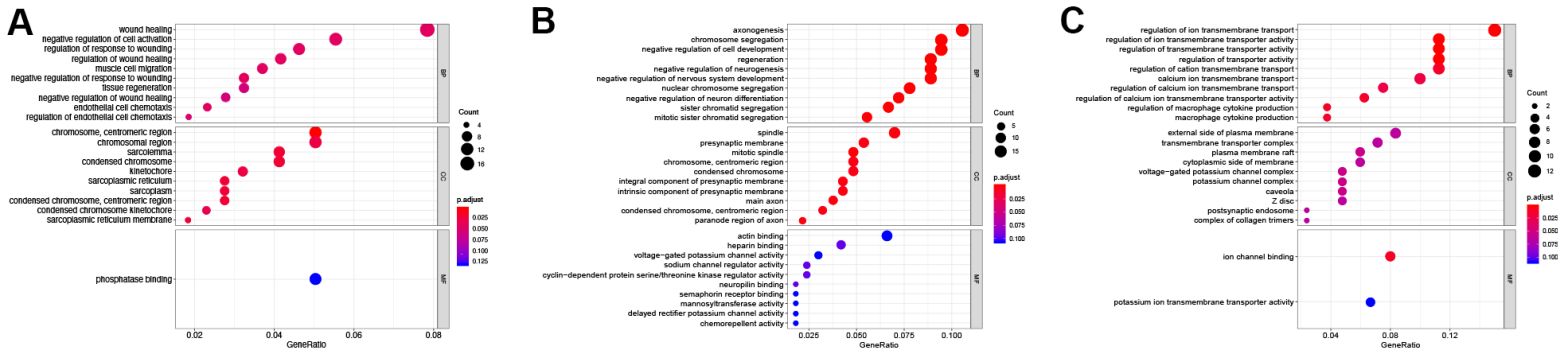
**GO enrichment of differentially expressed lncRNAs.** (**A**) GO enrichment of DElncRNAs unique to the control vs D4 group. (**B**) GO enrichment of common DElncRNAs shared by the control vs D4 and control vs D7 groups. (**C**) GO enrichment of DElncRNAs unique to the control vs D7 group.

**Figure 5:**
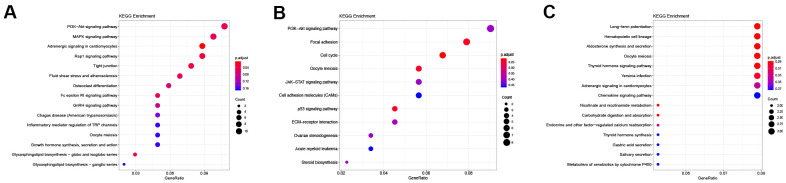
**KEGG enrichment of differentially expressed lncRNAs.** (**A**) KEGG enrichment of DElncRNAs unique to the control vs D4 group. (**B**) KEGG enrichment of common DElncRNAs shared by the control vs D4 and control vs D7 groups. (**C**) KEGG enrichment of DElncRNAs unique to the control vs D7 group.

The cis- and trans-target genes (Supplementary Materials) were identified on the DElncRNAs unique to the control vs D4 group, the DElncRNAs unique to the control vs D7 group, and the common DElncRNAs in these two groups. After intersecting the DEGs in the corresponding groups, these target genes were subjected to GO and KEGG pathway enrichment analysis to better understand the function of the DElncRNAs in each group. The screening criteria were set as follows: the enrichment p value was less than 0.05 (note that the legends in Figures 4, 5 display the adjust p value).

The results of GO enrichment analysis showed that in BP the DElncRNAs unique to the control vs D4 were mainly enriched in wound healing, regulation of response to wound and endothelial cell chemotaxis and other GO terms related to wound healing; in CC, these lncRNAs were mainly enriched in cellular regions related to chromatin, centriole and sarcoplasmic reticulum; and they were only enriched in a single GO term, phosphatase binding, in MF (Figure 4A and Table 2). The common DElncRNAs shared by control vs D4 and control vs D7 groups were mainly enriched in axongenesis, chromosome segregation and regeneration and other biological processes related to axon regeneration and cell cycle; in CC, they were mainly enriched in spindle, presynaptic membrane and main axon and other regions; in MF, they were enriched in actin binding, heparin binding and sodium and potassium ion channel activity (Figure 4B and Table 3). The DElncRNAs unique to the control vs D7 group were mainly enriched in ion transmembrane transporter channel activity in BP; these lncRNAs were mainly enriched in transmembrane transport complex and ion channel complex in CC; and they were mainly enriched in ion channel binding and potassium ion transmembrane transporter activity in MF (Figure 4C and Table 4).

**Table 2:** GO functional enrichment of DElncRNAs unique to the control vs D4 group.

**Ontology**	**ID**	**Description**	**GeneRatio**	**P value**	**Predicted gene symbol**
BP	GO:0042060	wound healing	17/218	4.32E-05	Ubash3b, Smad3, Plau, Dusp10, Myof, Vim, Pdgfa, Anxa1, Ucn2, Epb41l4b, Plat, Plaur, Clec10a, Abat, Smoc2, Enpp4, Igsf10
BP	GO:0050866	negative regulation of cell activation	12/218	1.40E-05	Ubash3b, Rhbdd3, Cd37, Tnfaip8l2, Fcgr2b, Tnfrsf14, Lst1, Pdgfa, Anxa1, Inpp5d, Abat, Mnda
BP	GO:1903034	regulation of response to wounding	10/218	8.76E-05	Ubash3b, Smad3, Plau, Dusp10, Pdgfa, Epha4, Anxa1, Abat, Smoc2, Enpp4
BP	GO:0061041	regulation of wound healing	9/218	5.89E-05	Ubash3b, Smad3, Plau, Dusp10, Pdgfa, Anxa1, Abat, Smoc2, Enpp4
BP	GO:0014812	muscle cell migration	8/218	8.91E-05	Camk2d, Plau, Met, Retn, Pdgfa, Anxa1, Plat, Trib1
CC	GO:0000775	chromosome, centromeric region	11/220	1.02E-05	Nek2, Ska1, Aurka, Mki67, Cenpu, Cdca8, Cenpt, Wdhd1, Dsn1, Incenp, Cenpn
CC	GO:0098687	chromosomal region	11/220	0.00073947	Nek2, Ska1, Aurka, Mki67, Cenpu, Cdca8, Cenpt, Wdhd1, Dsn1, Incenp, Cenpn
CC	GO:0042383	sarcolemma	9/220	0.00027609	Prx, Camk2d, Ryr2, Casq1, Mlip, Dtna, Kcnb1, Anxa1, Sgcd
CC	GO:0000793	condensed chromosome	9/220	0.00043882	Nek2, Ska1, Aurka, Mki67, Cenpu, Cenpt, Dsn1, Incenp, Cenpn
CC	GO:0000776	kinetochore	7/220	0.00073353	Nek2, Ska1, Cenpu, Cenpt, Dsn1, Incenp, Cenpn
MF	GO:0019902	phosphatase binding	10/200	0.00029987	Smad3, Magi2, Met, Nek2, Vim, Pstpip1, Ghr, Gab2, Ppp1r9a, Ppp1r18

**Table 3:** GO functional enrichment of the common DElncRNAs shared by the control vs D4 and control vs D7 groups.

**Ontology**	**ID**	**Description**	**GeneRatio**	**P value**	**Target gene symbol**
BP	GO:0007409	axonogenesis	19/180	3.65E-07	Gdnf, Sema3e, Sema4g, Lgr6, Mbp, Pmp22, Ctnna2, Ret, Sema6c, Dixdc1, Chn1, Dclk1, Nfasc, Map2, Ptprz1, Slit3, Runx3, Tnr, Nexn
BP	GO:0007059	chromosome segregation	17/180	1.74E-08	Mki67, Incenp, Ccnb1, Top2a, Nusap1, Tacc3, Ska1, Kif14, Ncapg, Hjurp, Rmi2, Kif23, Fam83d, Pttg1, Ccne1, Cdk5rap2, Hecw2
BP	GO:0010721	negative regulation of cell development	17/180	8.55E-07	Dusp10, Sema3e, Sema4g, Ccnb1, Mbp, Pmp22, Sox2, Erbb4, Sema6c, Dixdc1, Ldlr, Kank1, Map2, Cdk5rap2, Ptprz1, Tnr, Ptbp1
BP	GO:0031099	regeneration	16/180	1.61E-07	Mki67, Dusp10, Ucn2, Ccnb1, Lgr6, Lif, Ccna2, Cd9, Sox2, Erbb4, Ccne1, Cdkn1a, Socs3, Rapgef3, Lifr, Tnr
BP	GO:0050768	negative regulation of neurogenesis	16/180	5.83E-07	Dusp10, Sema3e, Sema4g, Mbp, Pmp22, Sox2, Erbb4, Sema6c, Dixdc1, Ldlr, Kank1, Map2, Cdk5rap2, Ptprz1, Tnr, Ptbp1
CC	GO:0005819	spindle	13/186	3.51E-05	Incenp, Ccnb1, Ckap2l, Nusap1, Tacc3, Ska1, Rmdn2, Kif14, Kif23, Fam83d, Dlgap5, Cdk5rap2, Hecw2
CC	GO:0042734	presynaptic membrane	10/186	7.38E-05	Kcna1, Kcna2, Ctnna2, Cadm3, Erbb4, Gpm6a, Btbd8, P2ry2, Adra1a, Pcdh17
CC	GO:0072686	mitotic spindle	9/186	2.47E-06	Ckap2l, Nusap1, Tacc3, Ska1, Rmdn2, Kif23, Fam83d, Cdk5rap2, Hecw2
CC	GO:0000775	chromosome, centromeric region	9/186	8.53E-05	Nup133, Mki67, Incenp, Ccnb1, Ska1, Ncapg, Cenpa, Hjurp, Cenph
CC	GO:0000793	condensed chromosome	9/186	0.00012615	Nup133, Mki67, Incenp, Ccnb1, Top2a, Ska1, Ncapg, Cenpa, Hjurp
MF	GO:0003779	actin binding	11/167	0.00243689	Ermn, Ctnna2, Anln, Dixdc1, Flnc, Parvg, Hook1, Mprip, Map2, Ncald, Nexn
MF	GO:0008201	heparin binding	7/167	0.00097639	Lgr6, Nav2, Lamc2, Fgf7, Crispld2, Slit3, Fgf12
MF	GO:0005249	voltage-gated potassium channel activity	5/167	0.0017631	Kcnk1, Kcng1, Kcna1, Kcna2, Hcn1
MF	GO:0017080	sodium channel regulator activity	4/167	0.00024297	Scn3b, Snta1, Fxyd2, Fgf12
MF	GO:0016538	cyclin-dependent protein serine/threonine kinase regulator activity	4/167	0.00090138	Ccnb1, Ccna2, Ccne1, Cdkn1a

**Table 4:** GO functional enrichment of DElncRNAs unique to the control vs D7 group.

**Ontology**	**ID**	**Description**	**GeneRatio**	**P value**	**Target gene symbol**
BP	GO:0034765	regulation of ion transmembrane transport	12/80	1.81E-06	Stac, Rapgef3, Hecw2, Kcnq4, Nos1ap, Gstm2, Fhl1, Clic5, Kcnab1, Cabp1, Atp1b1, Kcnd3
BP	GO:0032412	regulation of ion transmembrane transporter activity	9/80	2.50E-06	Stac, Rapgef3, Hecw2, Nos1ap, Gstm2, Fhl1, Kcnab1, Cabp1, Atp1b1
BP	GO:0070588	calcium ion transmembrane transport	8/80	9.68E-05	Stac, Rapgef3, Nos1ap, Slc24a2, Gstm2, Plcb1, Cabp1, Atp1b1
BP	GO:1903169	regulation of calcium ion transmembrane transport	6/80	0.00011035	Stac, Rapgef3, Nos1ap, Gstm2, Cabp1, Atp1b1
BP	GO:0010935	regulation of macrophage cytokine production	3/80	3.38E-05	Cd74, Cuedc2, Wnt5a
CC	GO:0009897	external side of plasma membrane	7/84	0.00222475	Cd74, Cr2, Lepr, Cd24, Adgre1, Cd8a, Ssc5d
CC	GO:1902495	transmembrane transporter complex	6/84	0.00356396	Kcnq4, Nos1ap, Clic5, Kcnab1, Atp1b1, Kcnd3
CC	GO:0044853	plasma membrane raft	5/84	0.00032547	Nos1ap, Lipe, Atp1b1, Cd8a, Kcnd3
CC	GO:0098562	cytoplasmic side of membrane	5/84	0.00278281	Stac, Kcnab1, Rgs8, Cabp1, Rps28
CC	GO:0008076	voltage-gated potassium channel complex	4/84	0.00057464	Kcnq4, Nos1ap, Kcnab1, Kcnd3
MF	GO:0044325	ion channel binding	6/75	4.28E-05	Stac, Rapgef3, Fhl1, Kcnab1, Cabp1, Kcnd3
MF	GO:0015079	potassium ion transmembrane transporter activity	5/75	0.00082826	Kcnq4, Slc24a2, Kcnab1, Atp1b1, Kcnd3

KEGG pathway enrichment results (Table 5) showed that the DElncRNAs unique to the control vs D4 group were mainly enriched in the PI3K-Akt signaling pathway, MAPK signaling pathway and Rap1 signaling pathway (Figure 5A). The common DElncRNAs of these two groups were mainly enriched in the PI3K-Akt signaling pathway, focal adhesion and the cell cycle (Figure 5B). Interestingly, the control vs D4 group unique DElncRNAs and common DElncRNAs were both enriched in the PI3K-Akt signaling pathway. However, the specific DElncRNAs were totally different in these two groups: the control vs D4 unique DElncRNAs that were enriched in the PI3K-Akt signaling pathway included Col4a5, Fgf1, Ghr, Magi2, Met, Pdgfa, Ppp2r2b, Ppp2r2c, Prkaa2, Prlr and Thbs2, while the common DElncRNAs enriched in this pathway included Ccne1, Cdkn1a, Col9a1, Erbb4, Fgf7, Itgb8, Lamc2 and Tnr. The DElncRNAs unique to the control vs D7 group were mainly enriched in the long-term potentiation, hematopoietic cell lineage and aldosterone synthesis and secretion pathways (Figure 5C).

**Table 5:** KEGG pathway enrichment of the DElncRNAs unique to the control vs D4, the control vs D7 and the common DElncRNAs shared by these two groups.

**Group**	**ID**	**Description**	**GeneRatio**	**P value**	**Target gene symbol**
control vs D4 unique	rno04151	PI3K-Akt signaling pathway	11/102	0.00166352	Col4a5, Fgf1, Ghr, Magi2, Met, Pdgfa, Ppp2r2b, Ppp2r2c, Prkaa2, Prlr, Thbs2
control vs D4 unique	rno04010	MAPK signaling pathway	10/102	0.00167384	Cacna1e, Cd14, Dusp10, Dusp5, Fgf1, Map2k6, Mapk11, Mapk13, Met, Pdgfa
control vs D4 unique	rno04261	Adrenergic signaling in cardiomyocytes	9/102	3.74E-05	Camk2d, Ppp2r2c, Scn7a, Ryr2, Ppp2r2b, Adcy1, Mapk11, Mapk13, Myh7
control vs D4 unique	rno04015	Rap1 signaling pathway	9/102	0.00062217	Adcy1, Fgf1, Magi2, Map2k6, Mapk11, Mapk13, Met, Pdgfa, Rap1gap
control vs D4 unique	rno04530	Tight junction	8/102	0.00042471	Cldn19, Cldn20, Epb41l4b, Nedd4l, Ppp2r2b, Ppp2r2c, Prkaa2, Rab13
common	rno04151	PI3K-Akt signaling pathway	8/89	0.01971696	Ccne1, Cdkn1a, Col9a1, Erbb4, Fgf7, Itgb8, Lamc2, Tnr
common	rno04510	Focal adhesion	7/89	0.00348967	Col9a1, Flnc, Itgb8, Lamc2, Mylk, Parvg, Tnr
common	rno04110	Cell cycle	6/89	0.00128807	Ccna2, Ccnb1, Ccne1, Cdkn1a, Pkmyt1, Pttg1
common	rno04114	Oocyte meiosis	5/89	0.00562757	Ar, Ccnb1, Ccne1, Pkmyt1, Pttg1
common	rno04630	JAK-STAT signaling pathway	5/89	0.0180481	Cdkn1a, Lif, Lifr, Pim1, Socs3
control vs D7 unique	rno04720	Long-term potentiation	3/38	0.00258342	Plcb1, Rapgef3, Rps6ka1
control vs D7 unique	rno04640	Hematopoietic cell lineage	3/38	0.00762447	Cd24, Cd8a, Cr2
control vs D7 unique	rno04925	Aldosterone synthesis and secretion	3/38	0.00783606	Atp1b1, Lipe, Plcb1
control vs D7 unique	rno04114	Oocyte meiosis	3/38	0.01252668	Ar, Fbxo5, Rps6ka1
control vs D7 unique	rno04919	Thyroid hormone signaling pathway	3/38	0.01397788	Atp1b1, Plcb1, Rcan2

### lncRNA-mRNA interaction network construction

**Figure 6:**
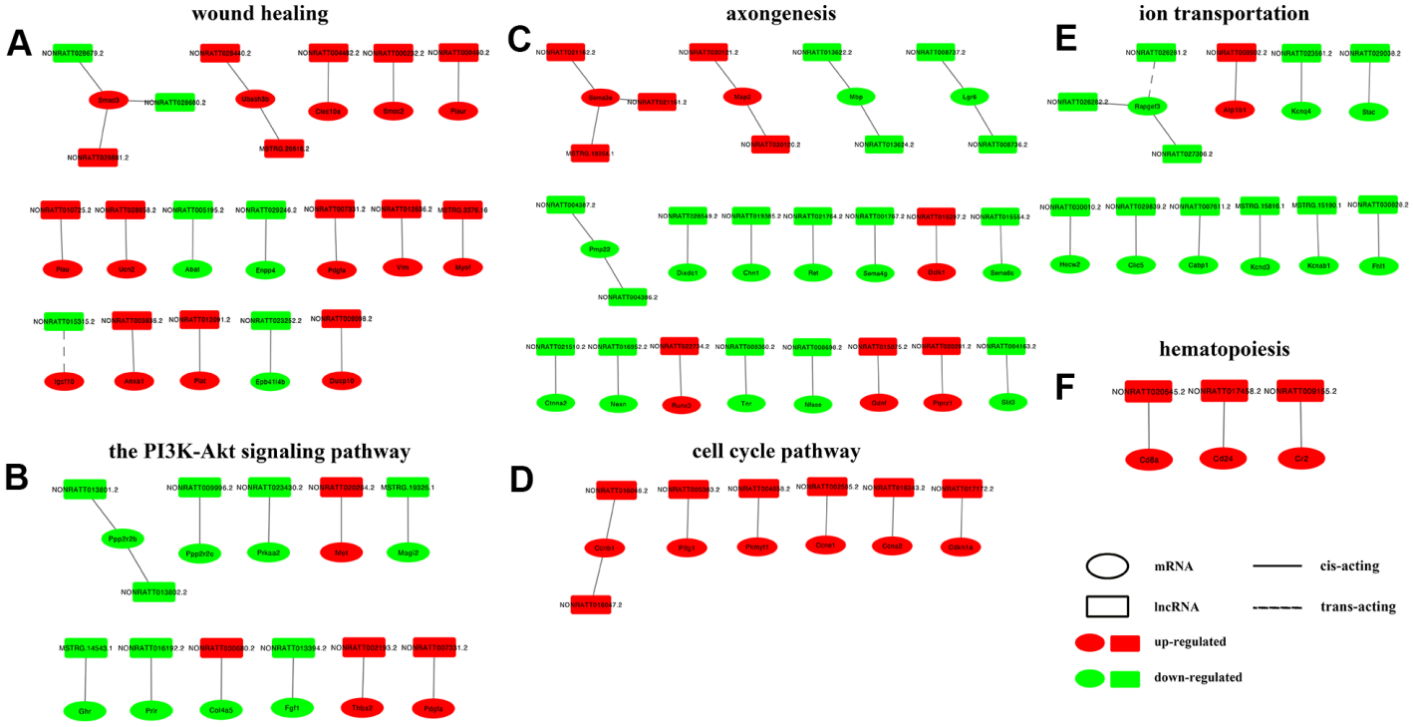
**Construction of lncRNA-mRNA networks.** DElncRNAs from top enriched GO terms and KEGG pathways and their target mRNAs were used to construct lncRNA-mRNA networks. In the network, upregulated mRNAs are displayed as red ellipses, downregulated mRNAs are displayed as green ellipses, upregulated lncRNAs are displayed as red rectangles, and downregulated lncRNAs are displayed as green rectangles. Solid lines connect lncRNAs and their cis-acting target genes, and dotted lines connect lncRNAs and their trans-acting target genes. (**A**) network constructed in GO term wound healing (top enriched GO term of DElncRNAs unique to the control vs D4 group). (**B**) network constructed in KEGG PI3K-Akt pathway (top enriched KEGG pathway of DElncRNAs unique to the control vs D4 group). (**C**) network constructed in GO term axongenesis (top enriched GO term of common DElncRNAs). (**D**) network constructed in KEGG cell cycle pathway (top enriched KEGG pathway of common DElncRNAs). (**E**) network constructed in GO term ion transportation (top enriched GO term of DElncRNAs unique to the control vs D7 group). (**F**) network constructed in KEGG hematopoiesis pathway (top enriched KEGG pathway of DElncRNAs unique to the control vs D7 group).

The GO terms and KEGG pathways with high enrichment scores and strong correlations with nerve regeneration were selected, and lncRNA-mRNA interaction networks were constructed within each group, with the regulation trend and the interaction mechanism annotated. The lncRNA-mRNA interaction networks of wound healing and the PI3K-Akt pathway, highly enriched GO term and KEGG pathway of the control vs D4 group unique DElncRNAs are displayed in Figure 6A, 6B, respectively. The networks of axongenesis and cell cycle pathways, highly enriched GO term and KEGG pathway of common DElncRNAs are displayed in Figure 6C, 6D, respectively. The networks of ion transportation and hematopoiesis, highly enriched GO term and KEGG pathway of the control vs D7 group unique DElncRNAs are displayed in Figure 6E, 6F, respectively. From the interaction networks, we found the following rules: (1) most lncRNAs are consistent with their corresponding mRNA regulation trends, and the expression trends of only a few pairs of lncRNAs and mRNAs are opposite, including NONRATT028679.2-Smad3, NONRATT028680.2-Smad3, NONRATT015315.2-Igsf10 and NONRATT030680.2-Col4a5; (2) the action mode of most lncRNAs on mRNAs is cis-acting, except NONRATT015315.2-Igsf10 and NONRATT026281.2-Rapgef3, whose mode of action is trans-acting; (3) the regulatory relationship between most lncRNAs and mRNAs is one-to-one, only a few mRNAs are regulated by two lncRNAs, and very few mRNAs are regulated by three lncRNAs at the same time.

### RT-qPCR verification

**Figure 7:**
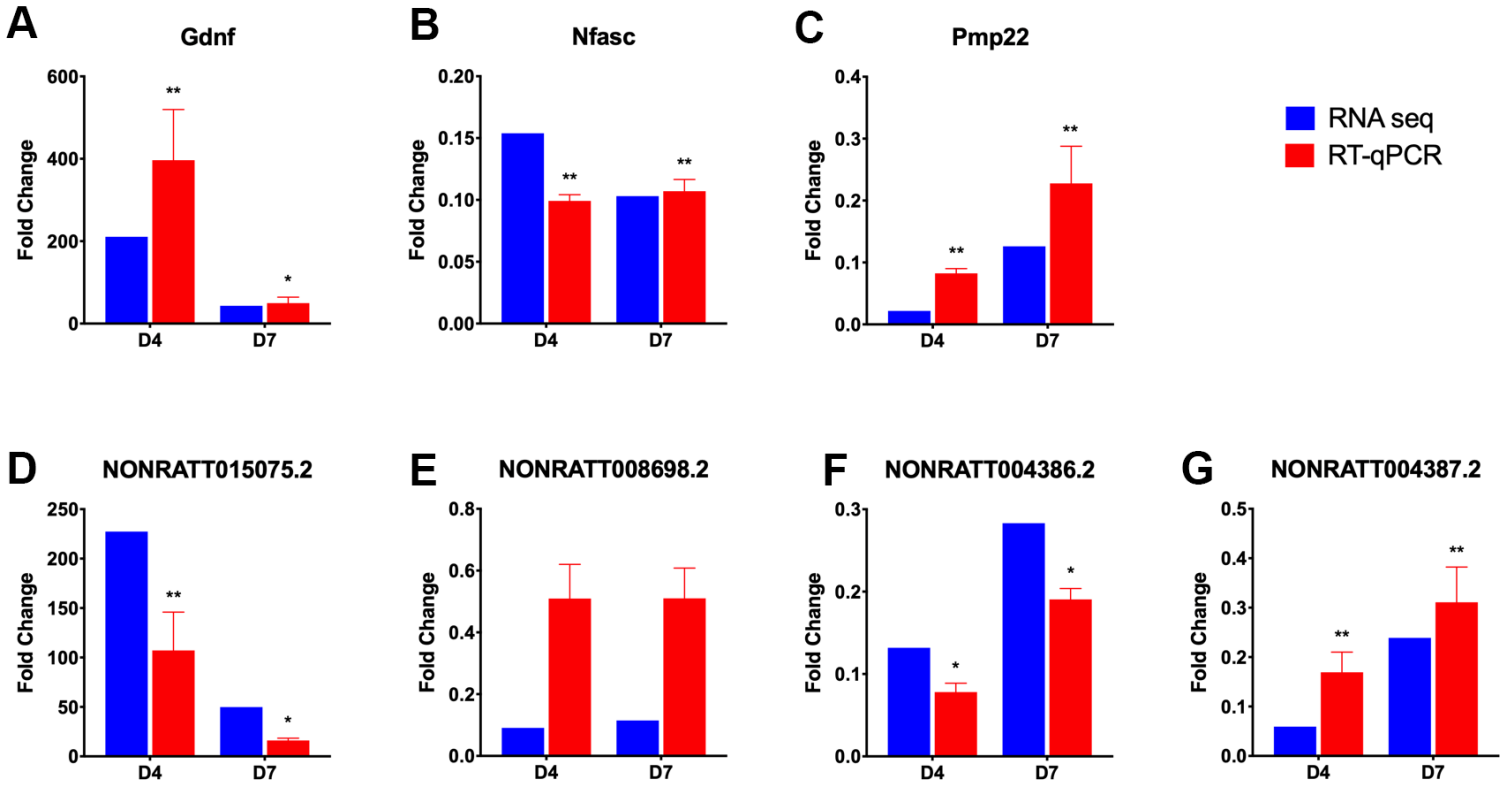
**RT-qPCR verification of selected differentially expressed lncRNAs and mRNAs.** Three lncRNA-mRNA interaction pairs were selected from the lncRNA-mRNA network constructed in axongenesis GO term, and their expression, expressed as fold change value compared with control group (omitted), were verified using RT-qPCR (red bar). The lncRNA-mRNA pairs selected are Gdnf (**A**) and NONRATT015075.2 (**D**); Nfasc (**B**) and NONRATT008698.2 (**E**); Pmp22 (**C**), NONRATT004386.2 (**F**) and NONRATT004387.2 (**G**). And the results were compared with those obtained from RNA-seq (blue bar). Statistically significant values are presented as asterisks (*), *P < 0.05, **P < 0.01.

Three sets of lncRNA-mRNA interaction pairs enriched in GO term axongenesis (Figure 6C), including 3 mRNAs and 4 lncRNAs, namely, Gndf and NONRATT015075.2, Nfasc and NONRATT008698.2, Pmp22 and NONRATT004387.2, NONRATT004386.2 were selected, and their expression was measured using RT-qPCR to verify the accuracy of the RNA-seq sequencing results. The RT-qPCR results (Figure 7) showed that the expression trends of the above 7 genes on D4 and D7 post-operation compared to the control group were completely consistent with the RNA-seq results. In addition to the NONRATT008698.2 gene, the expression levels of the other 6 genes on D4 and D7 all had significant differences compared to the control group.

## DISCUSSION

After peripheral nerve injury, the injured distal axon undergoes Wallerian degeneration, accompanied by a phenotypic change in Schwann cells from dedifferentiation (to guide axon regeneration) to redifferentiation (to promote the myelination of regenerated axons) [23], and this process involves a variety of biological activities from gene transcription and epigenetic regulation [24, 25]. Although an increasing number of studies have attempted to clarify the role of a single gene in this process in recent years, the regulatory relationship among genes is still not clear. In this study, with the use of RNA-seq techniques and bioinformatics methods, we identified the differentially expressed lncRNAs, predicted their potential role in the early post-PNI stage, and finally constructed the lncRNA-mRNA interaction networks in the pathways or biological processes in which those lncRNAs were enriched.

In our study, 579 common DElncRNAs were shared by the control vs D4 and control vs D7 groups, which participate in the whole process of the regulation of gene expression in the early stage after sciatic nerve injury. These lncRNAs were mainly enriched in axongenesis, chromosome segregation, regulation of cell development, spindle, presynaptic membrane and ion channel activity in GO enrichment. In KEGG enrichment analysis, these DElncRNAs were mainly enriched in the PI3K-Akt signaling pathway, focal adhesion, JAK-STAT signaling pathway, the ECM-receptor interaction pathways. These GO terms or KEGG pathways that DElncRNAs were mainly enriched were highly consistent with the results of the DEG functional enrichment analysis in rat sciatic nerve injury reported by previous studies [26–28], indicating a high credibility of evidence that the lncRNAs we analyzed have the potential to play a role in the regulation of PNI-associated genes. To determine the different functions of the DElncRNAs at different time points after injury, we analyzed the GO and KEGG enrichment of DElncRNAs unique to the control vs D4 and control vs D7 groups. The results showed that the unique DElncRNAs in the control vs D4 group were mainly enriched in wound healing, regulation of wound healing and endothelial cell chemotaxis-related GO terms, suggesting that the regulatory effect of lncRNAs on injured nerve repair and angiogenesis may begin in the very early stages after nerve injury. The DElncRNAs unique to the control vs D7 group were mainly enriched in GO terms related to ion transmembrane transport, especially channels related to calcium and potassium, which play important roles in axon degeneration and nerve repair. After PNI, the influx of extracellular calcium and the release of intracellular calcium can induce the activation of axon proteases, such as calpains, which in turn cause Wallerian degeneration at the distal end of the injury [29]. Studies have shown that the reduction in intracellular calcium can lead to the delayed occurrence of axon degeneration [30]. Studies on potassium channels in PNI have shown that Kv2.1 ion channels on the surface of motor neurons rapidly decrease one week after injury and then gradually recover [31], although the influence of such a change is not clear. Interestingly, we found that the DElncRNAs unique to the control vs D4 group and the common DElncRNAs were both highly enriched in the PI3K-Akt signaling pathway. However, the mRNAs regulated by DElncRNAs of the two are totally different: the control vs D4 unique DElncRNAs mainly regulate the Col4a5, Fgf1, Ghr, Magi2, Met, Pdgfa, Ppp2r2b, Ppp2r2c, Prkaa2, Prlr and Thbs2 genes in the PI3K-Akt signaling pathway, while the common DElncRNAs mainly regulate the expression of Ccne1, Cdkn1a, Col9a1, Erbb4, Fgf7, Itgb8, Lamc2 and Tnr genes in this pathway. The PI3K-Akt signaling pathway is considered to be closely related to peripheral nerve axon growth, myelination, and Schwann cell function [32]. Our findings suggested that the different components of the PI3K-Ak signaling pathway may be regulated by different lncRNAs at different stages after PNI.

GO terms and KEGG pathways with high enrichment scores were selected and the lncRNA-mRNA interaction networks were then constructed within these terms and pathways. In the PI3K-Akt signaling pathway, enriched by the DElncRNAs unique to the control vs D4 group, there are two sets of lncRNA-mRNA interaction networks that attracted our attention: NONRATT013801.2/NONRATT013802.2-Ppp2r2b and NONRATT009996.2-Ppp2r2c. Among them, Ppp2r2b and Ppp2r2c belong to the regulatory B subunit of protein phosphatase 2A (PP2A). Akt is the main substrate of PP2A, and inhibition of PP2A can enhance the activity of Akt [33]. Combined with the results of our study: the expression levels of Ppp2r2b and Ppp2r2c on D4 post injury, including their regulatory lncRNAs, were downregulated. We speculated that lncRNAs NONRATT013801.2 and NONRATT013802.2 could inhibit the expression of PP2A, leading to the upregulation of Akt and its downstream mTOR signaling pathway, finally activating the expression of c-Jun, a key factor that promotes Schwann cells entering the repair state [34, 35].

In the GO term axonogenesis enriched by the common DElncRNAs, lncRNA NONRATT015075.2 may lead to the upregulation of the glial cell-line-derived growth factor (Gdnf) gene. Gdnf, a TGF-β-related neurotrophic factor, can promote the proliferation and migration of Schwann cells and help the regeneration of neuronal axons [36–38]. Sema3e (semaphorin 3e) and its receptor play an important role in axonal guidance [39]. In this study, we predicted 3 lncRNAs that could regulate Sema3e, namely, NONRATT021162.2, NONRATT021161. 2 and MSTRG.19354.1. In the GO term ion transmembrane transport, enriched by the control vs D7 unique DElncRNAs, we noticed that 3 pairs of lncRNA-mRNA were all related to voltage-gated potassium channels. They were NONRATT023561.2 and Kcnq4, MSTRG.15816.1 and Kcnd3, MSTRG.15190.1 and Kcnab1. The expression of the above 3 pairs of genes all showed a downward-regulated trend. Similarly, Zhao et al. found that lncRNA Kcna2 antisense RNA can cause the downregulation of voltage-gated potassium channel Kcna2, which may be related to neuralgia after PNI [40]. Finally, we selected 3 groups of lncRNA-mRNA interaction pairs, a total of 7 genes including 3 mRNAs and 4 lncRNAs, and used RT-qPCR to detect their expression levels. The results showed that the results of RT-qPCR and RNA-seq results were completely consistent. However, the regulatory relationship between lncRNAs and their target genes needs further study.

Studies have shown that aging and its comorbidities, such as hypertension and diabetes, have an important impact on the plasticity of both central and peripheral nerve systems after injury [41–43]. However, in the current study, as we did not include the aging animals as our study objects, it is unable for us to clarify the impact of senescence on the lncRNAs expression profile after peripheral nerve injury, which is the limitation of this study and needs us to further investigate in the future studies.

In this study, we used NGS technology to sequence rat sciatic nerve tissues on D4 and D7 after injury, analyzed the DElncRNAs at different time points, and predicted the target genes combined with the corresponding mRNA expression data. The DElncRNAs at different time points were analyzed for functional enrichment, and the GO terms and KEGG pathways with high enrichment rates were selected to construct the lncRNA-mRNA interaction networks. This study preliminarily revealed the regulatory role of lncRNAs at different time points in the early stage after PNI, which could provide new potential targets for the research and treatment of PNI.

## Supplementary Material

Supplementary Table 1

Supplementary Table 2

Supplementary Table 3
