# Supplementary material for: Integrated analysis of long noncoding RNAs and mRNA expression profiles reveals the potential role of lncRNAs in early stage of post-peripheral nerve injury in Sprague-Dawley rats
Source: Aging (Albany NY). 2021 May 8;13(10):13909–25. doi: 10.18632/aging.202989 (PMC8202893; doi:10.18632/aging.202989)
Supplement: Supplementary Table 3 [file aging-13-202989-s003.pdf]

SUPPLEMENTARY TABLE

Supplementary Table 3. Primer sequences of candidate and reference genes.

Gene name	Primer sequences
Gdnf-F	AGGCCAGGCATGTTGCA
Gdnf-R	CCAGGCTGTCGTCTAAAAACG
NONRATT015075.2-F	TTCAGAGGTGTTCTGTTGTAGAATGA
NONRATT015075.2-R	GCCTCTCCCTTTCAGCTCTTC
Nfasc-F	TGGGAGAAGATATCCAGAGTTTTGT
Nfasc-R	GGTCACCTGATCTGCTGCTACA
NONRATT008698.2-F	GCCTGGCCAAGAGAGCTATG
NONRATT008698.2-R	CACTCCCTGCTCCTCCTTCTG
Pmp22-F	GAGCATCAGGACGAGCGTCTA
Pmp22-R	GACCCAGAAAGCCAGGGAAT
NONRATT004387.2-F	CCTCACACCCTCCTTCCTCTC
NONRATT004387.2-R	GAACAGGATCCCCAACAAGAGT
NONRATT004386.2-F	CATCCTTCAGGAAACTCCTCTCC
NONRATT004386.2-R	GATCCCCAACAAGAGTAGAAGCA
Gapdh-F	TGGCCTCCAAGGAGTAAGAAAC
Gapdh-R	GGCCTCTCTCTTGCTCTCAGTATC
